# Modeling of Nonlinear Aggregation for Information Fusion Systems with Outliers Based on the Choquet Integral

**DOI:** 10.3390/s110302426

**Published:** 2011-02-25

**Authors:** Kuo-Lan Su, You-Min Jau, Jin-Tsong Jeng

**Affiliations:** 1 Department of Electrical Engineering, National Yunlin University of Science & Technology, 123 University Road, Section 3, Douliou, 64002 Yunlin, Taiwan; 2 Graduate School of Engineering Science and Technology, National Yunlin University of Science & Technology, 123 University Road, Section 3, Douliou, 64002 Yunlin, Taiwan; E-Mail: g9510809@yuntech.edu.tw; 3 Department of Computer Science & Information Engineering, National Formosa University, Wunhua Road, Huwei Township, 64632 Yunlin, Taiwan; E-Mail: tsong@nfu.edu.tw

**Keywords:** information fusion, multi-sensor systems, Choquet integral, particle swarm optimization with quantum-behavior, least trimmed squares

## Abstract

Modern information fusion systems essentially associate decision-making processes with multi-sensor systems. Precise decision-making processes depend upon aggregating useful information extracted from large numbers of messages or large datasets; meanwhile, the distributed multi-sensor systems which employ several geographically separated local sensors are required to provide sufficient messages or data with similar and/or dissimilar characteristics. These kinds of information fusion techniques have been widely investigated and used for implementing several information retrieval systems. However, the results obtained from the information fusion systems vary in different situations and performing intelligent aggregation and fusion of information from a distributed multi-source, multi-sensor network is essentially an optimization problem. A flexible and versatile framework which is able to solve complex global optimization problems is a valuable alternative to traditional information fusion. Furthermore, because of the highly dynamic and volatile nature of the information flow, a swift soft computing technique is imperative to satisfy the demands and challenges. In this paper, a nonlinear aggregation based on the Choquet integral (NACI) model is considered for information fusion systems that include outliers under inherent interaction among feature attributes. The estimation of interaction coefficients for the proposed model is also performed via a modified algorithm based on particle swarm optimization with quantum-behavior (QPSO) and the high breakdown value estimator, least trimmed squares (LTS). From simulation results, the proposed MQPSO algorithm with LTS (named LTS-MQPSO) readily corrects the deviations caused by outliers and swiftly achieves convergence in estimating the parameters of the proposed NACI model for the information fusion systems with outliers.

## 1. Introduction

In the modern world, to make optimum decisions in economics, industry, science, aeronautics, manufacturing, traffic control, and many other military and civilian applications we are extremely dependent on useful and crucial information which is drawn from messages or data via transformation, classification and/or some other processing. Therefore, multi-sensor systems providing these messages or data are becoming increasingly important in meeting the goals of optimum decision-making. Besides, a feasible model to elaborate on information fusion and a soft computing technique to perform the heavy computations required are also critical.

Within the consideration of a feasible model, traditionally, the most common forms are the weighted average model and the linear regression model. These models are all linear and assume that there is no interaction among feature attributes (*i.e*., input information). However, in many real-world systems, the inherent interaction among feature attributes must be considered circumspectly and these kinds of systems are essentially non-additive systems. Hence, a nonlinear aggregation based on a nonlinear integral (NANI) model with respect to a non-additive set function is a powerful way of coping with these kinds of systems. In general, the Choquet integral is the most frequent form of the nonlinear integral and some literature proposing its use exists [1–4]. Liu *et al*. [1] proposed a NACI model derived from one of the following three kinds of fuzzy supports: the bespoke fuzzy support, the sample relative fuzzy support and the response correlative fuzzy support. This model deals with the interaction among feature attributes based on the correlation in statistics. Wang *et al*. proposed the original [2] and weighted [3,4] NACI model to deal with the information with numerical and categorical feature attributes, respectively. In fact, the weighted NACI model is the generalized form of the original one. In these two models, the interaction among the feature attributes toward the objective attributes (*i.e*., outputs) is described as non-additive set functions and is essentially derived from the co-relationship in the statistics. Although the weighted NACI model is successful in describing the interaction among hybrid feature attributes, at the same time, more parameters have to be estimated than in the original NACI model, but for a system with *n*-dimensional feature attributes, there are 2*^n^* + *n* parameters that must be determined and it is obvious that the amount of parameters increases exponentially with the dimensions of the feature attributes. The problem of exactly finding out these parameters is an essential optimization problem and the basic idea consists of making the residuals as small as possible. Residuals here are defined as the difference between what is actually observed and what is estimated. To minimize residuals, traditionally, the Least Square (LS) method is introduced and typically it achieves a remarkable estimation under circumstances where all attributes are uncontaminated. Unfortunately, in real world applications these features and objective attributes are always subject to outliers. That is, outliers may occur due to various reasons, such as erroneous measurements or data with a heavy-tailed distribution function. Whenever outliers exist, they always cause a serious deviation of what is estimated. Within the outlier detection literature [5–7], the least trimmed squares (LTS) estimator and the least median squares (LMS) estimator are the most popular ways of eliminating the effects caused by outliers. The LTS estimator not only possesses a high breakdown value but also several advantages over the LMS estimator, therefore, in this study we have focused our efforts on the LTS estimator to eliminate the inference from outliers. That is, we propose a feasible model able to effectively reject outliers that is also a contribution of this paper to the fuzzy integral problem.

Confirming the feasible model and from previous analysis, to efficiently and swiftly estimate the model’s parameters satisfying specific criteria is the next challenge. That is, a timesaving soft computing technique is necessary for the information fusion system with contaminated attributes. In the literature, there are many outstanding soft computing techniques that qualify for this task; they are neural network (NN) [8], GA [9], ant colony optimization (ACO) [10], *etc*. Particle swarm optimization with quantum-behavior (QPSO) which is an improved version of the traditional particle swarm optimization (PSO) [11] would be one of the powerful choices [12–13]. In the QPSO algorithm, particles are bounded in the searching range just like electrons move in a quantum well; meanwhile, according to the uncertainty principle, a particle’s position and velocity cannot be determined simultaneously. Hence, the information of a particle in quantum space is depicted by probabilities (*i.e*., wave function) and the dynamic behavior of a particle is widely divergent and dominated by the Schrödinger equation. The QPSO algorithm ensures the congregation of the particle swarm without losing the randomness. Within the QPSO algorithm, particles can appear at any position of the whole space which is searched with a certain probability. This algorithm offers high performance in single mode systems, because of the property of swift convergence. However, particles usually fall into local extreme states in multimode optimization systems and then take on the premature phenomenon. In order to make use of the merits of quick convergence and conquer premature in the traditional PSO, we proposed a QPSO algorithm with elitist crossover mechanism of the GA (named MQPSO) in our previous work [14] and demonstrated a superior performance than the GA in estimations of model parameters. In this paper, we improve the MQPSO algorithm proposed in our previous work to manipulate systems with outliers. That is, the mechanism of the LTS estimator is introduced to eliminate deviations caused by outliers and enhance the robustness of the MQPSO algorithm. To distinguish it, the revised MQPSO algorithm is named LTS-MQPSO. The most significant improvement is that the LTS-MQPSO algorithm combines the concepts of the simulated annealing (SA) and the GA within the QPSO algorithm to achieve global search and overcome prematurity in optimal processes, respectively; meanwhile, the LTS estimator is also performed to eliminate the inference from outliers. In order to verify the proposed LTS-MQPSO algorithm, a numerical example is also performed in this study. From the results of the experiment, the proposed LTS-MQPSO algorithm is able to acquire reasonable parameters for the NACI model and make quite precise decisions.

The rest of paper is organized as follows: in Section 2, we introduce the NACI model and characterize the information fusion system. Section 3, the least trimmed square estimator and the QPSO algorithm are briefly described. Next, we propose the LTS-MQPSO algorithm in detail. Section 5, is shown the results of numerical simulation and then the paper is concluded in Section 6.

## 2. The NACI Model and Information Fusion System Characterization

In traditional linear aggregations, the most frequent model used to describe the relation between feature attributes *X* and objective attribute *Y* is the Lebesgue-like integral [15]:

(1) $$Y=\kappa_{0}+\kappa_{s}\cdot \int \mathrm{fd}\upsilon +\mathrm{er}$$

where *κ*_0_ is a constant, *κ_s_* is a scaling factor, the integrand *f* represents observations of the scope of feature attributes *X*, *υ* is an additive measure which indicates the relative contribution of each element of feature attributes and *er* is the error term which has the form of normally distributed random perturbation with zero mean and variance *σ*^2^. This linear model always performs a good approximation based on a fundamental assumption that there is no interaction among feature attributes. However, in many real-world systems, the inherent interaction among feature attributes must be considered circumspectly. To reasonably describe the inherent interaction among feature attributes, Wang and Klir [16,17] proposed a regular non-additive set function *μ* named normalized general measure (NGM). The NGM is defined on the power set of feature attributes and the formal definition of the NGM can be express as:

(2) μ(Ø)=0,  μ(X)=1, when Ø∈P(X)

(3) A∈P(X), B∈P(X), and A⊂B⇒μ(A)≤μ(B)

Besides, a nonlinear integral is also introduced to aggregate the feature attributes. That is, whenever we deal with information fusion systems where information possesses some inherent interactions, the nonlinear integral with respect to the NGM is the most reasonable tool. In practical applications, there are many kinds of nonlinear integrals such as the Choquet integral [18], the Sugeno integral [19], the Wang integral [20], and so on. The Sugeno integral, by definition, is similar to logical operations and thus it is not an extension of the Lebesgue-like integral. Although the Sugeno integral is very timesaving to perform, it cannot be precisely inverted and this is a fatal defect. On the other hand, the Wang integral has been shown to possess remarkable properties. However, it is rather complex and quite time-consuming to perform. Those are the main reasons why the Choquet integral is adopted in this paper. The Choquet integral with respect to the NGM is defined as follows:

(4) $$\int \mathrm{fd}\;\mu =\int_{0}^{\infty }\mu (F_{\alpha })d\alpha$$

where *f*, {*f*(*x*_1_), *f*(*x*_2_),⋯*f*(*x_n_*)} is a non-negative measureable function with *n*-dimensions on *X*, and *F_α_* = {*x*|*f*(*x*) ≥ *α*, *x* ∈ *X*}, *α* ∈ [0, ∞), is called the *α*-cut set of function *f*. Since *X* is a finite set and the value of measureable function *f* can be sorted as:

(5) $$\underset{1\leq i\leq n}{\text{min}}\;f(x_{i})=f(x_{1}^{*})\leq f(x_{2}^{*})\leq \cdots \leq f(x_{n}^{*})=\underset{1\leq i\leq n}{\text{max}}\;f(x_{i})$$

where $\left\{x_{1}^{*},x_{2}^{*},\cdots ,x_{n}^{*}\right\}$ is a permutation of {*x*_1_,*x*_2_,⋯,*x_n_*}. Then, the discrete type of Choquet integral with respect to the NGM defined above can be expressed as:

(6) $$\int \mathrm{fd}\;\mu =\sum_{i=1}^{n}\left(f(x_{i}^{*})-f(x_{i-1}^{*})\right)\cdot \mu (\left\{x_{i}^{*},x_{i+1}^{*},\cdots ,x_{n}^{*}\right\})\;\text{with}\;f(x_{0}^{*})=0$$

Compared to the linear aggregation model shown in Equation (1), *μ*({*x_i_*}) represents the relative strength of contribution to objective attributes *Y* by a single feature attribute *x_i_*, and *μ*(*A*), *A* ∈ *P*(*X*) represents the joint relative strength of contribution to objective attributes *Y* by the feature attribute set *A*. In addition, to simultaneously deal with observations with categorical attributes and numerical attributes, the NACI model which indicates the relation between hybrid attributes *X* and objective attributes *Y* can be expressed by the following formula [4]:

(7) y=c+q⋅∫(ω⋅f)d μ+er

where *c* and *q* are constants, ∫ *fd μ* is the Choquet integral of function *f* with respect to the NGM *μ*, vector *ω* = (*ω*_1_,*ω*_2_,⋯,*ω_n_*) is an *n*-dimensional weighting vector which is used for coping with categorical attributes, *i.e.*, vector *ω* = (*ω*_1_,*ω*_2_,⋯,*ω_n_*) is used for balancing the units among various attributes and satisfies the following constraint:

(8) $$0<\omega_{i}\leq 1\;\text{and}\;\underset{1\leq i\leq n}{\text{max}}\omega_{i}=1,\;i=1,2,\cdots ,n$$

In the NACI model, constants *c*, *q*, vectors *ω* and the NGM *μ* are all parameters of the model. In total there are 2*^n^* + *n* unknown parameters and this number increases exponentially with the dimensions of the feature attributes. In order to complete the NACI model, these model’s parameters have to be determined in advance. That is so called the training state of the NACI model. In the training, associating Equation (7) with available observations constitutes an over-determined system with the Choquet integral. Thus, the analytic solution of the model parameters cannot be figured out exactly. Furthermore, constants *c* and *q* are essentially different from the other parameters which are governed by the Choquet integral. Therefore, a dual optimization procedure must be simultaneously performed; meanwhile, the performance index of optimization *J* (called fitness function) is also introduced and expressed as:

(9) $$J=\text{Minimize}\;\left\{e^{2}\right\}=\text{Minimize}\;\left\{\sum_{j=1}^{k}{\left(y_{j}-c-q\cdot \int (\omega \cdot f_{j})d\;\mu \right)}^{2}\right\}$$

where *k* is the length of available observations for the training state.

**Figure 1.**
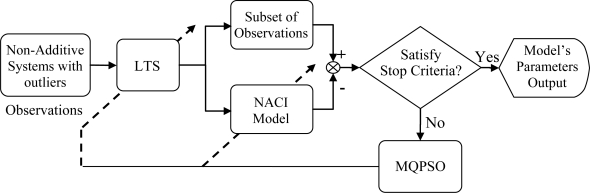
Block diagram of the proposed structure for the parameters estimation of the NACI model via MQPSO and LTS in the training state.

**Figure 2.**
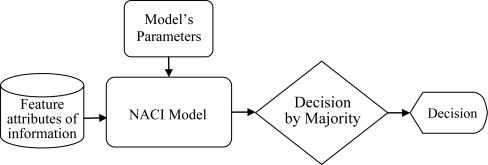
Block diagram of the proposed structure for information fusion systems.

Because the kernel of the performance index of optimization is the LS estimator, it always suffers from atypical observations which arise from outliers in real world systems. That is, the LS method deviates seriously in estimations of a model’s parameters where outliers are present. Hence, it is also a major objective of this study to propose a feasible method for resolving this issue. The proposed method has to achieve not only precise model’s parameters but also remarkable capability of rejecting outliers. In general, these kinds of problems are also called robust regressions and many high breakdown value regression estimators have been proposed for this [6,7]. For the reasons of simplicity and efficiency, the LMS and the LTS are the more popular regression estimators in scientific applications. Furthermore, the LTS estimator possesses not only the same breakdown value as the LMS, but also several additional merits: for instance, its objective function is smoother; its statistical efficiency is better, and so on. Therefore, we focus the treatment of outliers in the LTS method and thus, Equation (9) is revised as:

(10) $$J=\text{Minimize}\;\left\{\sum_{j=1}^{h}{\left(y_{j}^{*}-c-q\cdot \int (\omega \cdot f_{j}^{*})d\;\mu \right)}^{2}\right\}$$

where $y_{j}^{*}$ and $f_{j}^{*}$ are a permutation of observations under the best model parameters and *h* is a trimmed parameter of the LTS estimator. The block diagram of the proposed structure for the training state and information fusion systems is shown in Figures 1 and 2, respectively.

In Figure 1, the block named MQPSO receives the differences of objective attributes between observations and estimations when the terminative criterion is not satisfied yet; meanwhile, the parameters of the NACI model are updated based on these differences. Another block which is named LTS is used for filtering out these atypical observations and the trimmed parameter of the LTS estimator *h* is also revised by the global optimal parameters so far. Besides, the block named “Non-additive systems with outliers” is the system that we are considering. That is, it is the source of the training data (Observations) which are used for modeling the NACI. The block named “Subset of observations” is represented as the observations after the LTS. That is, the “Subset of observations” is also „Non-additive systems’ but different from the Observations (Non-additive systems with outliers). In Figure 2, the block named feature attributes of information depicts continued observations in a period in which the decision profile (DP) is produced. Associating the DP with the model’s parameters which are acquired in the training state, the decision is usually able to be made precisely. Besides, the block named decision by majority guarantees that we are always able to make a correct decision in a low contaminated environment.

## 3. The LTS Estimator and the QPSO Algorithm

The LTS estimator is formulated as:

(11) $$\text{Minimize}\;\left\{\sum_{i=1}^{h}r^{2}(d_{i})\right\}\;\text{and}\;r^{2}(d_{1})\leq r^{2}(d_{2})\leq \cdots \leq r^{2}(d_{h})\leq \cdots \leq r^{2}(d_{k})$$

where *d_i_* is the *i_th_* observation, *r*^2^ (*d_i_*) is the *i_th_* squared residual, *k* is the length of observations and *h* is the number of data points which are not trimmed from the data set. In robust regression analysis [6], the maximum tolerance of the LTS estimators to outliers (named Maximum Breakdown Point) for any equivariant regression estimator satisfies:

(12) $$\text{Breakdown Point}\leq \frac{1}{k}((\left(k-\zeta \right)/2)+1)$$

where *ζ* is the dimension of variables. Intuitively, the breakdown point is bounded above at 50%. The maximum breakdown point is actually attained for Equation (12) with *h* = (*k* + *ζ* +1)/2 in a multiple regression system and the solution of Equation (11) always exists. Of course, one can achieve the optimal solution by considering $C_{h}^{k}$ ordinary least squares problems for all subsets of {1,2,⋯,*k*} with *h* elements and selecting the best one among all candidates. Obviously, it is laborious and impractical for real world systems with large numbers of observations. In order to cope with a great deal of observations, the FAST-LTS method was been proposed [7]. The major distinguishing features are the initial *h*-subset, the C-step and the nested extensions. By and large, the initial *h*-subset is a preselecting mechanism to confirm that a clean *h*-subset {*d*_1_,*d*_2_,⋯,*d_h_*} drawn from all observations can be attained. The C-step is a recursive procedure and used for increasing the accuracy of the estimated model parameters. This recursive procedure estimates a model parameters *θ_ini_* with the LS estimator based on a clean *h*-subset $\left\{d_{1}^{*},d_{2}^{*},\cdots ,d_{h}^{*}\right\}$ which is created by the initial *h*-subset procedure. Then, the newly square residuals and *h*-subset $\left\{d_{1}^{\mathrm{new}},d_{2}^{\mathrm{new}},\cdots ,d_{h}^{\mathrm{new}}\right\}$ are acquired in turn. By the new *h*-subset, the estimated *θ_new_* is more accurate than *θ_ini_*. Repeating these procedures, a set of precise model parameters *θ* can be achieved. For a small to moderate data size *k*, these two procedures work well and do not take much time. When the number of observations is large enough, for instance *k* > 600, the performance of these two procedures is poor and it takes much more time. To deal with this situation, the procedure named nested extension is introduced. In nested extensions, the data is partitioned into many subsets and then, the initial *h*-subset and the C-step are applied to each subset. Next, each subset with *λ* feasible solutions is extended to the full observations and the C-step procedure performed repeatedly. Finally, an optimal solution that satisfies the specific desired accurateness would be achieved.

After drawing observations without contaminations, a proper soft computing technique is essential and can help us to efficiently estimate the parameters of the NACI model. In the literature there are many outstanding soft computing techniques that qualify for this work. The QPSO algorithm is one of these soft computing techniques, and possesses significant global and local search abilities. In the QPSO algorithm, particles move in a quantum multi-dimensional space, the state of particles is usually depicted by normalized wave function Ψ(*ρ*,*t*), *i.e.*, the probability amplitude of the position where particles are present; and further, |Ψ(*ρ*,*t*)|^2^ is then interpreted as the corresponding probability density function which satisfies the follow equation:

(13) $$\int_{\begin{array}{l} \text{whole}\\ \text{space} \end{array}}{\left|\Psi (\rho ,t)\right|}^{2}d\;\rho =1$$

where *ρ* are the *n*-dimensional coordinates. That is, a single particle with mass *m* is subjected to the influence of a potential field *V*(*ρ*,*t*) in the quantum space and the wave function is governed by the Schrödinger equation:

(14) $$i\hbar \frac{\partial }{\partial t}\Psi (\rho ,t)=-\frac{\hbar^{2}}{2m}\nabla^{2}\Psi (\rho ,t)+V(\rho ,t)\Psi (\rho ,t)$$

where *ħ* is the Planck constant and ∇^2^ is the Laplacian operator. In an environment with a potential field, the particles are then attracted to the center of field through the optimization process, and this attraction leads to the global optimum. Based on the assumption that the attractive potential field is time-independent (the co-called stationary state), the solution of the time-independent Schrödinger equation has the form [21]:

(15) Ψ(ρ,t)=φ(ρ)⋅exp(−iωt)

where *ω* has the dimensions of an angular frequency. In theory, any type of potential well can describe this system which is bounded and attracted by a potential field. However, the simplest one is the Delta Potential Well and the potential field is given by:

(16) V(ρ)=−γδ(ρ)

where *γ* is a positive number proportional to the “depth” of the potential well. The meaning of Equation (16) is that the depth is infinite at the origin and zero elsewhere. For the sake of simplicity, the solution of time-independent Schrödinger equation for this system in one dimensional space is considered and expressed as:

(17) $$Q(z)={\left|\varphi (z)\right|}^{2}=\frac{1}{L}e^{-\frac{2\left|z\right|}{L}},L=\frac{\hbar^{2}}{m\gamma }$$

where *Q*(*z*) is the probability density function for measuring a particle’s state and *L* is the characteristic length of Delta Potential Well. The *L* specifies the search scope of a particle and is called “Creativity” or “Imagination”. In order to obtain the precise position of particles, the Monte Carlo Method is used for simulating the procedure whereby the quantum state collapses to the classic state. After this effort, the particle’s position can be expressed as:

(18) $$\varphi_{i}={\mathrm{pf}}^{\mathrm{cnt}}(i)\pm \frac{L}{2}\cdot \text{ln}\left(\frac{1}{u}\right),\;i=1,2,\cdots ,\mathrm{NP}$$

where *NP* is the number of particles in a population, *u* is random number uniformly distributed on [0,1] and *pf^cnt^* is the center of potential field which is proposed by Clerc and Kennedy [22] and defined as:

(19) $${\mathrm{pf}}^{\mathrm{cnt}}(i)=\frac{\left(c_{1}\cdot p_{i}^{\mathrm{loc}}+c_{2}\cdot p^{\mathrm{gol}}\right)}{\left(c_{1}+c_{2}\right)},\;i=1,2,\cdots ,\mathrm{NP}$$

where *c*_1_, *c*_2_ are constriction coefficients and $p_{i}^{\mathrm{loc}}$, *p^gol^* are the best position of the *i_th_* particle and the global best position found so far. In order to improve performance of the QPSO algorithm, Sun *et al*. [13] employ a Mainstream Thought Point (or named Mean Best Position, *mbest*) to evaluate the parameter *L*. However, to extend the global search of the QPSO algorithm, the *mbest* is modified and then, these two parameters can be expressed as the following form:

(20) $$\mathrm{mbest}=\left[\sum_{i=1}^{\mathrm{NP}}\frac{\phi_{i,1}}{\mathrm{NP}},\sum_{i=1}^{\mathrm{NP}}\frac{\phi_{i,2}}{\mathrm{NP}},\cdots ,\sum_{i=1}^{\mathrm{NP}}\frac{\phi_{i,n}}{\mathrm{NP}}\right]$$

(21) $$L=2\cdot \beta \left|\mathrm{mbest}-\varphi_{i}\right|$$

where *β* is a creative coefficient which is used to adjust the convergent speed of individual particle and the performance of the QPSO algorithm. Hence, the particle’s position can be updated in the each iteration by the form:

(22) $$\varphi_{i+1}={\mathrm{pf}}^{\mathrm{cnt}}(i)\pm \beta \left|\mathrm{mbest}-\varphi_{i}\right|\cdot \text{ln}\left(\frac{1}{u}\right)$$

## 4. The LTS-MQPSO Algorithm

**Figure 3.**
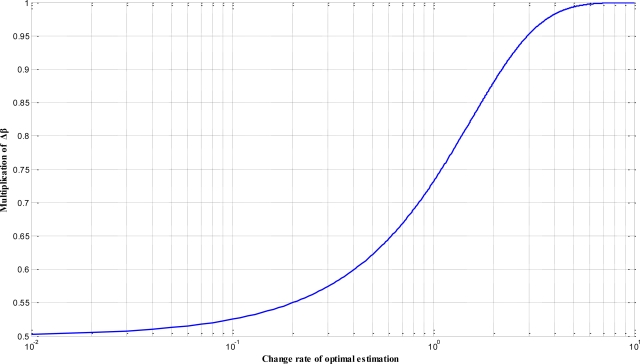
A typical curve of the creative coefficient *β* as affected by the changing rate of the optimal estimation Δ*fit*, where the traverse axis is a logarithmic scale.

Within empirical applications, however, the QPSO algorithm usually represents a stagnating phenomenon for searching the global optimal solution in multi-mode problems and systems. Meanwhile, it is also strongly influenced by the creative coefficient *β*. In order to improve these defects, the updating mechanism of the creative coefficient *β* on the MQPSO algorithm which is proposed in our previous works is revised. That is, the modified MQPSO algorithm combines the QPSO algorithm with mechanisms of the SA and the GA to achieve global search and overcome premature for traditional PSO in optimization process. Two significant improvements are introduced to the modified MQPSO algorithm. They are the nonlinear updating of the creative coefficient *β* with the form of the SA and the instantaneous monitoring the convergence of the optimization procedure, respectively. In the QPSO algorithm, the creative coefficient *β* is set to a large number at the beginning and adjusted decreasingly following the optimization procedure. Such mechanisms effectively realize that a global search is performed at the beginning and the convergence is achieved finally. In general, the decreasing rate of *β* is linear, but a nonlinear revision according to the convergence of the optimization process would be more reasonable and feasible. In the modified MQPSO algorithm, a nonlinearly revising mechanism which is similar to the SA algorithm is introduced and expressed as the form:

(23) $$\beta =\beta_{\mathrm{ini}}-\Delta \beta \cdot {\left(1+\text{exp}(-(\Delta \mathrm{fit}))\right)}^{-1}$$

where Δ*β* is step length of *β*, Δ*fit* is the changing rate of optimal estimation so far and *β_ini_* is the initial value of *β*. A typical curve of *β* which is adjusted by Δ*fit* is shown in Figure 3.

The other improvement of the modified MQPSO algorithm is the mechanism to overcome prematurity. Inspired by the mechanisms of mutation and elite crossover in the GA, an index of conquering stagnation (named *ECM* which is an abbreviation of Elite Crossover and Mutation) is used for monitoring the status of the optimization procedure in the modified MQPSO algorithm. That is, during the optimization procedure, the modified MQPSO algorithm preserves each different *p^gol^*; meanwhile, the index of conquering stagnation, *ECM* is set to zero whenever *p^gol^* is updated. Of course, the *ECM* increases by one whenever *p^gol^* is unchanged. Before finishing the current iteration, the modified MQPSO algorithm judges whether *ECM* exceeds the specific criteria. If it is true, the modified MQPSO algorithm lets the new population be these collected *p^gol^* instead of the original population (all/or these worse particles) and sets the *ECM* to zero, instantaneously.

**Figure 4.**
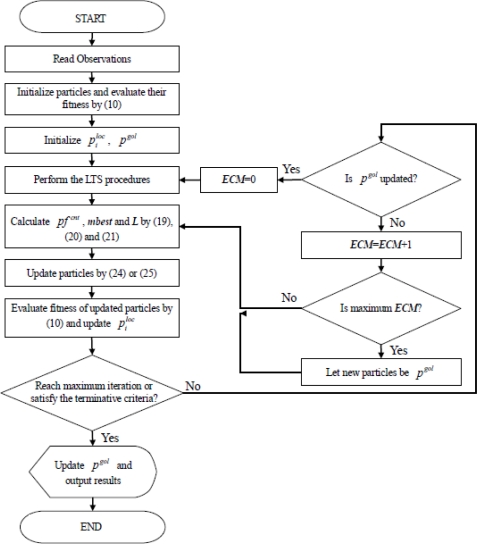
The flow chart of the proposed LTS-MQPSO algorithm.

For observations without outliers, the MQPSO algorithm offers superior performance for estimating parameters than the GA [14]. Because the kernel of estimating fitness is the LS estimator, the MQPSO algorithm always makes a serious deviation in the contaminated circumstance. Therefore, the LTS estimator is introduced to sieve out the observations without contamination. The proposed LTSMQPSO algorithm and flow chart is shown below and Figure 4.

**Step 1:** Randomly initialize the population of particles with dimension 2*^n^* − 2 + *n* and then, evaluate their fitness values by Equation (10).
**Step 2:** Sort particles according to their fitness values and then initialize $p_{i}^{\mathrm{loc}}$, *p^gol^*.
**Step 3:** Perform the LTS estimator to sieve out these *h* observations without contamination.
**Step 4:** Calculate *pf^cnt^*, *mbest* and *L* by (19), (20) and (21), respectively.
**Step 5:** Select (24) or (25) with randomly probability to update *φ_i_*:
  (24) $$\varphi_{i}(t+1)={\mathrm{pf}}^{\mathrm{cnt}}(i)-\beta \cdot {\mathrm{norm}}_{2}\left(\mathrm{mbest}-\varphi_{i}(t)\right)\cdot \text{ln}(\frac{1}{u})$$
  (25) $$\varphi_{i}(t+1)={\mathrm{pf}}^{\mathrm{cnt}}(i)+\beta \cdot {\mathrm{norm}}_{2}\left(\mathrm{mbest}-\varphi_{i}(t)\right)\cdot \text{ln}(\frac{1}{u}),$$
  where *norm*_2_(*ps*_1_ − *ps*_2_) denotes the distance between *ps*_1_ and *ps*_2_.
**Step 6:** Evaluate the fitness values of all particles base on (10).
**Step 7:** According to fitness values evaluated in Step 6, update $p_{i}^{\mathrm{loc}}$.
**Step 8:** Check over whether the maximum iteration is reached or the terminative criterion is satisfied? If yes, go to Step 11, else perform next Step.
**Step 9:** Check over whether *p^gol^* is updated? If *p^gol^* is updated, sets *ECM* to 0 and perform the LTS procedure, then go to Step 3. If *p^gol^* is unchanged, increase *ECM* by 1 and perform next Step.
**Step 10:** Check over whether the maximum *ECM* is reached? If yes, let these collected *p^gol^* instead of *φ*(*t* + 1) and go to Step 4, else keep *φ*(*t* + 1) and go to Step 4.
**Step 11:** Check over whether *p^gol^* should be updated and then output the results.

## 5. Numerical Simulation and Results

**Figure 5.**
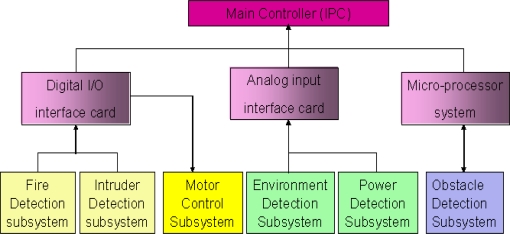
The hierarchy structure of the sensory systems used for the ISR.

The multi-sensor-based intelligent security robot (ISR) [23] consists of six subsystems; namely, sensor system, remote supervision system, software development system, image system, avoid obstacle and motion planning system. These subsystems can acquire and preliminarily processes sensory signals and then, the sensory data is transmitted by interface devices to the main controller (IPC) for further treatment. The hierarchy structure of sensory systems used for the ISR is shown in Figure 5. In the fire detection subsystem and intruder detection subsystem, the sensory data is transmitted by a digital input/output interface. That is, these two subsystems only send a decision which is made by an information fusion system to the IPC of the ISR. However, a wrong decision is usually made whenever the sensory signal is contaminated with outliers. In this simulation, we focus our attention on the fire detection subsystem. This subsystem is constituted by environmental sensors, which include flame sensors, smoke sensors and temperature sensors. It is suitable for demonstrating and verifying the effectiveness and feasibility of the proposed information fusion system shown in Figures 1 and 2. Prior to performing the numerical simulation, the principles of these three sensors are briefly described.

**Figure 6.**
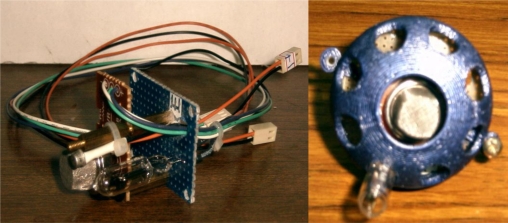
The 3-in-1 fire detection sensor used for the fire detection subsystem of the ISR.

In the smoke sensor module, the kernel is a TG135 ionization smoke sensor. When smoke occurs, an ionizing radioactive source is brought close to the plates and the air itself is ionized. In other words, it will generate a tiny current. For the flame sensory module, the R2868 ultraviolet sensor is used for detecting the flame. Its peak wavelength is 200 μm and its sensing wavelength is 185–260 μm. For the temperature sensory module, the AD590 semiconductor sensor is adopted to detect the temperature of fire. This sensor has a positive temperature coefficient of about 0.7, and its linearity is within 0.5% for a temperature range between −65 °C and 150 °C. The standard output of the AD590 is 1 mA/°K. In general, these sensory signals are all tiny values and have to be converted to a standardized voltage output by an amplifier circuit. Besides, the relations of input sensory signals and output voltage signals must be made linear by tuning the calibration circuits. Finally, these sensory signals that are converted to binary digital signals are transmitted to the IPC. In this experiment, these three modules are integrated together and the resulting 3-in-1 fire detection sensor is shown in Figure 6. Because the sensory signal is tiny, it always suffers from outliers and this causes a wrong output. Fortunately, these outliers only last an instant in general and we are able to eliminate them by considering the interactions among continuous samples. For the sake of simplicity, an artificial observation profile which simulates four continuous sampling data points with normalization is made to estimate the model’s parameters by associating the proposed LTS-MQPSO algorithm in the training state. All simulations are implemented in the Matlab environment and conducted on an Intel Core 2 Duo CPU P8400, 4GB Ram capacity PC.

**Example:** The original model parameters are set as: c = 5, q = 1.2, *ω* = {0.67 0.3 1 0.43}, *μ* = {0.2 0.12 0.35 0.4 0.56 0.5 0.6 0.3 0.45 0.38 0.6 0.73 0.9 0.83 1} and h = 75%. Then, we randomly create 400 4-dimensional feature attributes with 10% random contamination to produce training data as shown in Table 1, where *y_true_* are the original objective attributes *y_cont_* are the contaminated objective attributes and the bold-faced numbers represent that objective attributes are contaminated.

**Figure 7.**
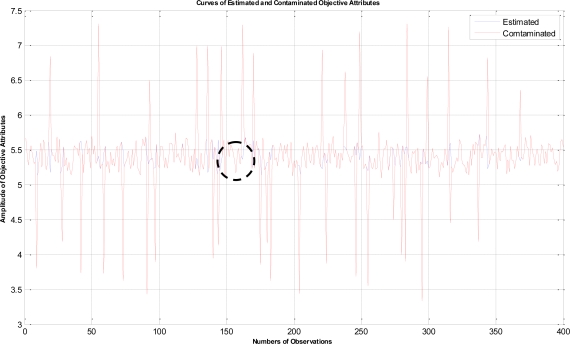
Shown the results for the contaminated objective attributes and the estimated objective attributes.

**Figure 8.**
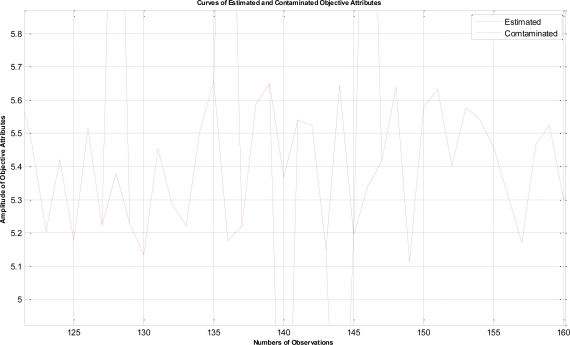
Zoom in the curve marked by a dotted circle in Figure 7.

**Figure 9.**
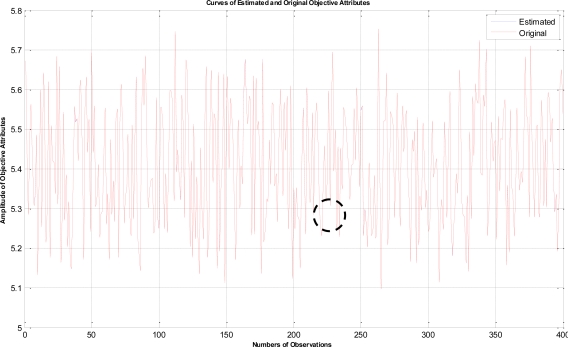
Shown the results for the original objective attributes and the estimated objective attributes.

**Figure 10.**
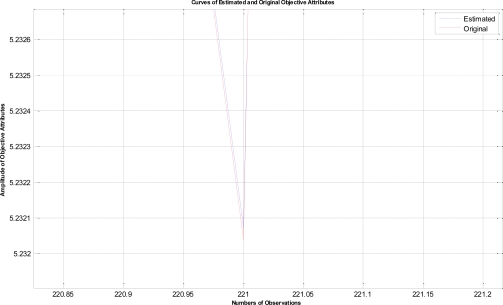
Zoom in the curve marked by a dotted circle in Figure 9.

In this example, the termination criteria of the program are that the iterations reach a maximum of 1,500 times or the mean square error is less than 10^−5^. After performing the proposed LTS-MQPSO algorithm for many times, the average results of estimating the model parameters and comparisons are shown in Tables 2–4. In addition, we also show in Figures 7–10 plots of the training data and estimated results. In Figure 7, a comparison between the contaminated (red line) and the estimated (blue dash line) objective attributes are shown. These two curves nearly overlap besides these points where outliers are present. To clearly show the performance of rejecting outliers, the zoomed in portion which is circled with a dotted line is also shown in Figure 8. As shown in Figure 8, the LTS-MQPSO algorithm is able to identify outliers and reject them. In the Figure 9, a comparison between the original (red line) and the estimated (blue dash line) objective attributes are shown. These two curves almost overlap everywhere. To distinguish each other, the zoomed in portion which is circled with dotted line is also shown in the Figure 10. As shown in this figure, the difference between the original and the estimated objective attributes is less than 10^−4^. Besides, it is intuitive that the LTS-MQPSO algorithm is able to make quite precise estimations of model’s parameters.

## 6. Conclusions

In this paper, the NACI model association with the LTS-MQPSO algorithm is considered and developed to deal with a non-additive system with outliers. Whenever atypical observations are present, the parameter estimation method based on the LS estimator is no longer feasible. Therefore, replacement of the LS estimator with the LTS estimator is an excellent alternative. That is, we successfully integrate the mechanisms of the SA, and the GA into the QPSO algorithm to estimate parameters of the NACI model; meanwhile, the LTS estimator is also introduced to filter out outliers before performing the modified MQPSO algorithm. From the simulation results, the proposed LTSMQPSO algorithm can precisely estimate parameters of the NACI model for observations contaminated with outliers; meanwhile, it still maintains high coincidence between the estimated and original objective attributes.

## Figures and Tables

**Table 1.** Tanning data with and without contaminations used for verifying the proposed NACI model and LTS-MQPSO algorithm.

	*x*_1_	*x*_2_	*x*_3_	*x*_4_	*y_true_*	*y_cont_*
01	0.760	0.900	0.790	0.930	5.67253	5.67253
02	0.930	0.210	0.440	0.260	5.39280	5.39280
03	0.680	0.850	0.070	0.750	5.28559	5.28559
04	0.260	0.940	0.900	0.030	5.49294	5.49294
05	0.860	0.790	0.630	0.690	5.56252	5.56252
06	0.670	0.120	0.420	0.710	5.41806	5.41806
07	0.190	0.760	0.460	0.210	5.30678	5.30678
08	0.920	0.180	0.400	0.040	5.33158	5.33158
09	0.650	0.680	0.450	0.920	5.48550	**3.80599**
10	0.050	0.710	0.210	0.010	5.13252	5.13252
11	0.290	0.640	0.600	0.290	5.39438	5.39438
12	0.270	0.040	0.940	0.650	5.60023	5.60023
13	0.970	0.400	0.080	0.290	5.25339	5.25339
14	0.450	0.460	0.890	0.810	5.64319	5.64319
15	0.730	0.020	0.910	0.330	5.58932	5.58932
16	0.650	0.120	0.160	0.210	5.21480	5.21480
17	0.490	0.790	0.150	0.910	5.31462	5.31462
18	0.530	0.900	0.820	0.720	5.62050	5.62050
19	0.170	0.580	0.070	0.620	5.17635	**6.84732**
20	0.870	0.820	0.790	0.030	5.50912	5.50912
⋮	⋮	⋮	⋮	⋮	⋮	⋮
385	0.820	0.120	0.250	0.680	5.35888	5.35890
386	0.480	0.240	0.160	0.200	5.19002	5.19003
387	0.960	0.110	0.830	0.140	5.55050	5.55042
388	0.880	0.370	0.210	0.660	5.35336	5.35336
389	0.710	0.650	0.350	0.330	5.34275	5.34284
390	0.420	0.840	0.130	0.430	5.23067	5.23077
391	0.680	0.650	0.390	0.400	5.36962	5.36974
392	0.330	0.320	0.810	0.640	5.55438	5.55440
393	0.650	0.790	0.340	0.710	5.40430	5.40440
394	0.840	0.030	0.250	0.580	5.34598	**6.35911**
395	0.300	0.940	0.480	0.520	5.39375	5.39379
396	0.770	0.770	0.200	0.810	5.36578	5.36589
397	0.520	0.980	0.760	0.010	5.44782	5.44787
398	0.580	0.920	0.870	0.900	5.68322	5.68323
399	0.790	0.910	0.720	0.370	5.53678	5.53670
400	0.370	0.560	0.890	0.210	5.52618	5.52621

**Table 2.** The average results of model parameters estimated by the proposed LTS-MQPSO algorithm.

**Parameters**	Estimated	Original	**Data**	Estimated	Contaminated	Original
*μ*_1_	0.202630	0.20	d(1)	5.67262	5.67253	5.67253
*μ*_2_	0.119595	0.12	d(2)	5.39267	5.39280	5.39280
*μ*_1,2_	0.352183	0.35	d(3)	5.28556	5.28559	5.28559
*μ*_3_	0.399180	0.40	d(4)	5.49297	5.49294	5.49294
*μ*_1,3_	0.561244	0.56	d(2)	5.56263	5.56252	5.56252
*μ*_2,3_	0.498894	0.50	d(6)	5.41794	5.41806	5.41806
*μ*_1,2,3_	0.601077	0.60	d(7)	5.30686	5.30678	5.30678
*μ*_4_	0.298500	0.30	d(8)	5.33147	5.33158	5.33158
*μ*_1,4_	0.451280	0.45	d(9)	5.48563	**3.80599**	5.48550
*μ*_2,4_	0.379223	0.38	d(10)	5.13246	5.13252	5.13252
*μ*_3,4_	0.601223	0.60	⋮	⋮	⋮	⋮
*μ*_1,2,4_	0.728169	0.73	d(391)	5.36962	5.36974	5.36974
*μ*_1,3,4_	0.900233	0.90	d(392)	5.55438	5.55440	5.55440
*μ*_2,3,4_	0.828266	0.83	d(393)	5.40430	5.40440	5.40440
*μ*_1,2,3,4_	1.000000	1.00	d(394)	5.34598	**6.35911**	5.34591
*ω*_1_	0.661194	0.67	d(395)	5.39375	5.39379	5.39379
*ω*_2_	0.299558	0.30	d(396)	5.36578	5.36589	5.36589
*ω*_3_	1.000000	1.00	d(397)	5.44782	5.44787	5.44787
*ω*_4_	0.430799	0.43	d(398)	5.68322	5.68323	5.68323
*c*	4.999999	5.00	d(399)	5.53678	5.53670	5.53670
*q*	1.202177	1.20	d(400)	5.52618	5.52621	5.52621

**Table 3.** The average results of objective attributes estimated by the LTS-MQPSO, the LTS-MQPSO-LB and the MQPSO algorithm.

**Data**	Original	Contaminated	Estimated by LTS-MQPSO	Estimated by LTS-MQPSO-LB	Estimated by MQPSO
d(1)	5.67253	5.67250	5.67253	5.67356	5.68501
d(2)	5.39280	5.39280	5.39280	5.39319	5.33520
d(3)	5.28559	5.28560	5.28559	5.28591	5.31600
d(4)	5.49294	5.49290	5.49294	5.49304	5.46360
d(2)	5.56252	5.56250	5.56252	5.56237	5.61565
d(6)	5.41806	5.41810	5.41806	5.42090	5.38345
d(7)	5.30678	5.30680	5.30678	5.30770	5.32974
d(8)	5.33158	**3.68524**	5.33158	5.33157	5.24508
d(9)	5.48550	5.48550	5.48550	5.48373	5.52952
d(10)	5.13252	5.13250	5.13252	5.13101	5.17121
d(11)	5.39440	5.39440	5.39438	5.39677	5.42818
d(12)	5.60020	5.60020	5.60020	5.60104	5.58952
d(13)	5.25340	5.25340	5.25353	5.25332	5.41922
d(14)	5.64320	5.64320	5.64318	5.64338	5.66970
d(15)	5.58930	5.58930	5.58939	5.58941	5.52346
⋮	⋮	⋮	⋮	⋮	⋮
d(386)	5.30790	5.30790	5.30781	5.309731	5.31757
d(387)	5.47460	5.47460	5.47471	5.476158	5.47223
d(388)	5.30380	5.30380	5.30377	5.303276	5.25468
d(389)	5.59290	5.59290	5.59300	5.594471	5.61154
d(390)	5.57300	5.57300	5.57296	5.573292	5.55016
d(391)	5.36974	5.46760	5.36974	5.464645	5.37275
d(392)	5.55440	5.52800	5.55440	5.529859	5.52020
d(393)	5.40440	5.38750	5.40440	5.387559	5.44484
d(394)	5.34591	5.29230	5.34591	5.29274	5.33559
d(395)	5.39379	**7.25114**	5.39379	5.337529	5.30333
d(396)	5.36589	5.19230	5.36589	5.192462	5.20087
d(397)	5.44787	5.30860	5.44787	5.307585	5.28206
d(398)	5.68323	5.61440	5.68323	5.614041	5.61254
d(399)	5.53670	5.65030	5.53670	5.651546	5.65586
d(400)	5.52621	5.53400	5.52621	5.533216	5.44066

**Table 4.** The average results of model parameters estimated by the LTS-MQPSO, the LTS-MQPSO-LB and the MQPSO algorithm.

**Parameters**	Original	Estimated by LTS-MQPSO	Estimated by LTS-MQPSO-LB	Estimated by MQPSO
*μ*_1_	0.20	0.202630	0.220264	0.999814
*μ*_2_	0.12	0.119595	0.101039	0.205399
*μ*_1,2_	0.35	0.352183	0.355931	0.000001
*μ*_3_	0.40	0.399180	0.446918	0.209780
*μ*_1,3_	0.56	0.561244	0.617110	0.000134
*μ*_2,3_	0.50	0.498894	0.542382	0.044604
*μ*_1,2,3_	0.60	0.601077	0.660831	0.002942
*μ*_4_	0.30	0.298500	0.249854	0.251532
*μ*_1,4_	0.45	0.451280	0.430170	0.000009
*μ*_2,4_	0.38	0.379223	0.337117	0.000087
*μ*_3,4_	0.60	0.601223	0.578540	0.289889
*μ*_1,2,4_	0.73	0.728169	0.718593	0.389799
*μ*_1,3,4_	0.90	0.900233	0.905735	0.347362
*μ*_2,3,4_	0.83	0.828266	0.827270	0.000376
*μ*_1,2,3,4_	1.00	1.000000	1.000000	1.000000
*ω*_1_	0.67	0.661194	0.689136	0.163266
*ω*_2_	0.30	0.299558	0.330364	0.163267
*ω*_3_	1.00	1.000000	1.000000	1.000000
*ω*_4_	0.43	0.430799	0.584927	0.269410
*c*	5.00	4.999999	4.999316	5.1152898
*q*	1.20	1.202177	1.075533	2.0519762
MSE		8.0154e-005	0.0018	0.455776
Elapse		1059 seconds	1496 seconds	1580 seconds
